# Maxillary Mucocele with Orbital Floor Remodelling

**DOI:** 10.1155/2012/439541

**Published:** 2012-08-08

**Authors:** Tahrina Salam, Maryam Zamani, Jane Olver

**Affiliations:** ^1^Moorfields Eye Hospital, London EC1V 2PD, UK; ^2^Ophthalmology Department, Charing Cross Hospital, London W6 8RF, UK

## Abstract

A 79-year-old man presents with signs of an orbital mass. A CT scan revealed a large maxillary mucocele eroding through the orbital floor. Surgical drainage of the mucocele and conservative postoperative care, returned all ophthalmic signs to normal and bony remodelling of the orbital floor was demonstrated. Maxillary mucoceles should be assessed by both ENT and Ophthalmic surgeons. Postoperative remodelling of the orbital floor can be illustrated with serial CT Scans.

## 1. Introduction 

Maxillary sinus mucoceles may present with varying symptoms. In severe cases ophthalmic signs may be the most prominent which need to be assessed pre- and postoperatively. Therefore a combined ENT and ophthalmic approach in assessing the patient and development of the management plan is essential.

## 2. Case Report

A-79-year old man presented to our tertiary oculoplastic and adnexal clinic with a five-month history of gradually increasing painless left proptosis associated with a reduction in visual acuity, double vision, swollen left cheek, and nasal congestion. The patient had no significant medical or family history. 

Visual acuity (VA) was 6/6 in the right eye and 6/18 in the left eye. Ishihara colour plates test was full at 17/17 in the right eye, and diminished to 12/17 in the left eye. No pupillary defect was noted. Optic disc appeared normal on dilated fundoscopy. Exophthalmometry illustrated a 4 mm left-sided nonaxial proptosis. Diplopia was demonstrated in all positions of gaze, but was more pronounced on down gaze and adduction. Nasal endoscopy revealed normal mucosa; however his left nostril was obstructed by the medialisation of the medial antral. There was no apparent lymphadenopathy on palpation or areas of numbness on his face.

**Figure 1:**
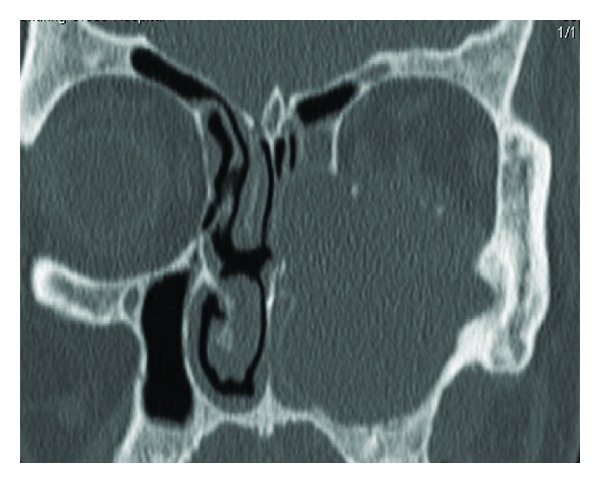
A coronal CT scan of a left maxillary mucocele eroding through the orbital floor and medial antral wall.

An urgent CT scan was performed showing a large maxillary mucocele eroding through the inferior orbital ridge and causing superolateral displacement of the globe. The medial antral wall was eroded with the mass projecting into the nasal cavity (Figure 1). There was no enhancing soft tissue to suggest the presence of malignancy.

Functional endoscopic sinus surgery was performed to drain the maxillary mucocele and 50 mL of thick yellow mucus was expressed, which was sent to pathology. This intervention induced an orbital decompression and preservation of optic nerve function. The mucosa appeared healthy and histology confirmed no evidence of malignancy.

**Figure 2:**
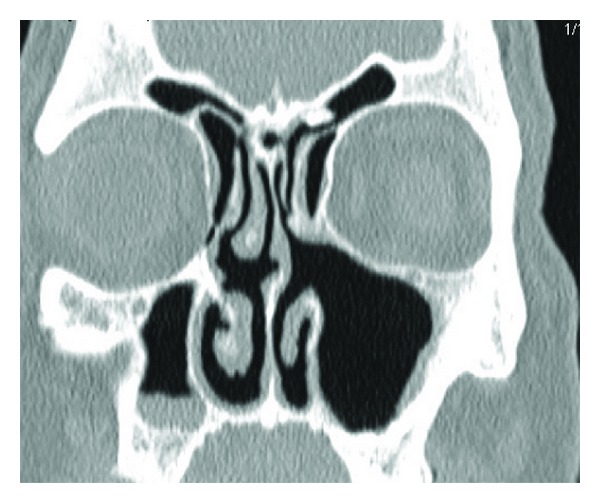
A coronal CT scan after ESS, showing remodeling of the orbital floor.

Within one month his left visual acuity and colour vision returned to normal with a VA of 6/6 bilaterally and 17/17 bilaterally with the ishihara colour test plates. He also had complete resolution of diplopia and proptosis. Postoperative CT scans show remodelling of the orbital floor (Figure 2) and therefore further reconstruction surgery to the orbital floor was not performed.

Two years later, the patient shows no evidence of recurrence of his mucocele. He has no ophthalmic symptoms or signs, and his occasional nasal symptoms are controlled with saline douches.

## 3. Discussion

Mucoceles are mucus-filled epithelial-lined lesions within sinus cavities. They can affect any paranasal sinus, and all can present with ophthalmic signs [1]. The most common paranasal sinuses affected are the frontal and ethmoidal sinuses (60% and 30%, resp.), followed by the more rarely affected maxillary sinus (which has a documented incidence of between 3% and 10%) and the sphenoid sinus [2, 3]. 

Mucoceles have a slowly expansile cystic nature that can cause sinus expansion, bony erosion, and invasion into nearby structures such as the orbit or nasopharynx [4]. Hence symptoms related to these mucoceles may have a gradual onset. The most common presenting complaints are facial pain, a palpable mass, proptosis, and diplopia [3–5]. A past medical history is essential in such patients as Khong et al. have shown that 93% of their patients had had previous sinus surgery and 80% of their patients had chronic sinusitis. Postoperative maxillary sinus mucoceles have been documented to present 10–19 years postsurgery [6, 7]. Other common causes of maxillary sinus mucoceles are chronic sinusitis and trauma. 

CT scan remains the investigation of choice, with mucoceles presenting as homogenous lesions with no contrast enhancement, unlike neoplasms. Bony walls may show some erosion, but clear margins will be present [7, 8].

Historically, treatment of paranasal mucoceles has involved removal of the mucosal lining and obliteration of the sinus. However, if invasion through the bone has already occurred with extension into the orbit, this procedure is not necessary. In these cases, removal of the mucosa from the orbit is difficult without damaging other orbital structures. If the sinus is then obliterated and a small amount of mucosa is still present in the orbit, recurrence of the mucocele is certain [8].

More recently, Endoscopic sinus surgery is favoured over a direct orbital approach as a safe and effective way of treating mucoceles with a lower morbidity, reduced hospital stay, and a mucocele recurrence rate from between 0.9%–8% [4, 9].

Our case demonstrates the need for a combined approach in assessing a patient with a maxillary sinus mucocele. Although ENT surgeons need to operate to remove the mucocele, ophthalmic assessment is essential in determining any evidence of optic nerve compression and the speed at which to progress to surgery. Our case is interesting as our patient did not have any typical symptoms of facial pain and sinusitis which may prompt a diagnosis of a mucocele. CT imaging at the time of presentation is essential in determining the amount of bony destruction the mucocele has caused and in planning the surgery. As illustrated by our case, even if marked bony destruction has occurred and there is gross extension through the orbital floor, this defect may be stabilized with postoperative scarring and bony remodelling. Therefore, assessment of the postoperative patient with the aid of CT imaging can help to manage the patient accordingly and prevent unnecessary surgery.
